# The Filth of the Middle Ages

**Published:** 1881-07

**Authors:** 


### THE FILTH OF THE MIDDLE AGES.

Dr. Lyon Playfair stated in his address at Glasgow, in 1874, that "when the civilization of the Egyptians, the Jews, the Greeks, and the Romans faded, the world passed through dark ages of mental and physical barbarism. For a thousand years there was not a man or woman who ever took a bath." "No wonder that there came the wondrous epidemics of the Middle Ages, which cut off one-fourth of the population of Europe." "But even when the Middle Ages had passed away and the sun of civilization was again rising over the gloomy darkness of the centuries, what a heritage of filth-producing diseases still remained!" Look at Montaigne's description of the plague at Bordeaux, from which he fled to his country house; it killed 18,000 out of 40,000 people, followed him, and destroyed whole villages; men's minds were occupied not with the thought of life, but how to protect their bodies from wild beasts after their death. He gives a terrible picture of one of his own workmen, whose last act was to draw the earth over his own expiring body. It is not a pleasing task to dwell on the habits of the population of England in past times. Go back only to the time previous to the Reformation, and you can have no difficulty in understanding why luxury and squalor produced the plagues of the times of the Tudors and Stuarts. The cabin of the peasant was made of reeds and sticks, and plastered over with mud. In these wigwams lived an ague-stricken population. In the towns the mechanics lived in rooms without glass windows, slept on straw beds, and worked in shops unheated by fire. Even in the well-to-do houses rushes covered the floor, and got saturated with scraps of food, which remained to putrify under new layers of rushes scattered over it, so that the "petre-man" came to dig saltpetre out of the floors. Filth, instead of being abhorred, was almost sanctified. The monks imitated the filthy habits of the hermits. St. Anthony never washed his feet; St. Thomas-a-Becket, when martyred, had undergarments in a state which makes one shudder in the remembrance. And so the monks, in part, up to the present day, thought, or pretended to think, that by antithesis pollution of the body indicated cleanliness of the soul. But this association of filth with religion was unhappy in its consequences, for men ceased to connect disease with uncleanliness, and resorted to shrines and winking Virgins for cures for maladies which were produced by their own physical and moral impurities.

For twenty years after the Restoration there was an exceptionally high mortality, even for that epoch, in the metropolis. Macaulay describes it as a time "when men died faster in the purest country air than they now do in the most pestilential lanes of our towns, and when men died faster in the lanes of our town than they now die on the coast of Guiana." Dr. Edward Bascombe, in his History of Epidemics, says that up to the year 1760, extraordinary as it may appear, there was not any such thing as a privy in Madrid; it was customary to throw the ordûre out of the windows at night, and it was removed by scavengers next day. An ordinance having been issued by the king that every householder should build a privy, the people violently opposed it as an arbitrary proceeding, and the physicians remonstrated against it, alleging that the filth absorbed the unwholesome particles of the air, which otherwise would be taken into the human body. His majesty, however, persisted, but many of the citizens, in order to keep their food wholesome, erected privies close to their kitchen fireplaces.—Adamson, *Medical Annals*, April, '81.

